# Supplementary material for: The Healthy Environments and Active Living for Translational Health (HEALTH) Platform: A smartphone-based system for geographic ecological momentary assessment research
Source: PLOS Digit Health. 2025 Dec 11;4(12):e0001133. doi: 10.1371/journal.pdig.0001133 (PMC12697974; doi:10.1371/journal.pdig.0001133)
Supplement: Attachment — Submitted filename: Response to Reviewers - HEALTH Protocol - 20250928.docx [file pdig.0001133.s001.docx]

**PDIG-S-25-00284 Response to Reviewers**

The following table includes our detailed responses to the reviewer reports received on July 21, 2025.

| **Reference** | **Reviewer Comment** | **Author Response** |
| --- | --- | --- |
| J1 | Please ensure that your Ethics Statement is available in its entirety at the beginning of your Methods section, under a subheading 'Ethics Statement'. | The Ethics Statement has been moved under a new subheading titled “2.0.1 Ethics Statement” at approximately Line 208. |
| J2 | Please upload separate figure files in .tif or .eps format. Also, remove the figures from your manuscript file but keep the legends. | We have removed all figures from the manuscript and uploaded as separate files in .tif format. |
| J3 | Some material included in your submission may be copyrighted. According to PLOS’s copyright policy, authors who use figures or other material (e.g., graphics, clipart, maps) from another author or copyright holder must demonstrate or obtain permission to publish this material under the Creative Commons Attribution 4.0 International (CC BY 4.0) License used by PLOS journals. Please closely review the details of PLOS’s copyright requirements here: PLOS Licenses and Copyright. If you need to request permissions from a copyright holder, you may use PLOS's Copyright Content Permission form. Please respond directly to this email or email the journal office and provide any known details concerning your material's license terms and permissions required for reuse, even if you have not yet obtained copyright permissions or are unsure of your material's copyright compatibility. Potential Copyright Issues: Figure 2: contains branding/a logo. We are not permitted to publish this under our CC-BY 4.0 license, even with permission. We ask that you please remove or replace it. Figures 2, 3, and 5: contains screenshots. We are not permitted to publish these under our CC-BY 4.0 license; websites are usually intellectual property and are copyrighted. This includes peripheral graphics of the web browser such as the [X] buttons. We ask that you please remove or replace it. | We have removed Figures 2, 3 and 5 as per Editorial instruction. |
| E1 | The reviewers found that the manuscript is well-conceived, methodologically sound, and highly relevant to the field of public health informatics and digital epidemiology. The HEALTH Platform is a meaningful contribution to mobile health interventions, with clear implications for behavioural surveillance, intervention personalisation, and real-time environmental exposure assessment. | Thank you for this positive assessment of our manuscript. |
| E2 | However, some sections, particularly in the discussion, would benefit from tighter narrative cohesion, clearer alignment with referenced literature, and more critical reflection on the limitations of the ParkSeek study.  Additionally, the reviewers suggest elaborating on privacy concerns, usability issues, and adding comparison with existing platforms. Please see their comments in detail below | Thank you for clarifying the direction we should take with our revisions. We have responded to the reviewers in detail below, making substantial improvements to the manuscript. |
| R1.1 | I generally found the HEALTH platform presented in this paper is innovative and technically superior and potentially could make it easier for the community to conduct GEMA studies. It addresses several challenges in current GEMA software platforms by having benefits including device-agnostics, update-free deployment and multiple data element support. The architecture appears to be robust, flexible and well explained. The ParkSeek Study is a solid study with interesting findings around survey prompt adherence. However, I did not find the presentation of the ParkSeek Study is able to fully demonstrate the flexibility of the mobile application and the capacity or advantage of meeting critical quality standard in GEMA field. Here are some comments. | Thank you for your feedback on the manuscript. We have provided detailed responses to your comments below. |
| R1.2 | The workflow for researchers: The paper does not sufficiently explain what the researcher setup process looks like, which is important to demonstrate the flexibility and usability of the platform. How could the ParkSeek survey be easily deployed to the platform without customized development? How customizable is it in practice for people with different skills? How easy is it to add different data type to the survey? I would recommend the paper takes some effort explaining this process using the ParkSeek Study | We have provided a general description of the workflow for researchers, and clarified the customizability of the HEALTH Platform to accommodate additional data types at approximately Line 183:  “The HEALTH Platform’s general workflow for the setup of a study consists of evaluating the data types to be collected by the HEALTH Platform app, designing the system instruction sets that will be applied to that data on the logic server, and the layout of the reporting tools provided in the monitor server. As further described in the following sections, the HEALTH Platform app is designed to be used with any web or smartphone-based application to provide researchers with the greatest flexibility in using their preferred data collection method or tool. The logic server makes use of a low-code development environment to configure the system instruction sets that are applied to collected data. The monitor server makes use of a similar low-code development environment to provide additional flexibility to researchers and practitioners in accessing reports about collected data. The HEALTH Platform is intended to provide an accessible and scalable system, leveraging open-source and freely available commercial software (for academic institutions), to undertake (G)EMA studies that do not rely on continual software development, ongoing system maintenance, or expensive subscription fees.” |
| R1.3 | Clarify how the platform impacts the cast study: The ParkSeek Study does not clearly articulate how the platform’s development contributed to solving specific challenges compared to previous tools or custom apps. What would have been difficult or impossible to achieve without the HEALTH platform? What specific problems faced by prior park exposure studies were overcome here? What kind of quality standard is met? A deeper analysis would strengthen the case that HEALTH is not only technically superior but practically transformative. | We have clarified the purpose of the cast study at approximately Line 347:  “The purpose of the following case study is to illustrate the potential of the HEALTH Platform for GEMA research. This example is not intended to provide any actionable and meaningful findings relevant to the hypothesized relationship between park exposure and health. Instead, the following sections provide methodological findings that exemplify the HEALTH Platform’s improvements upon past (G)EMA methods. Moreover, the system instruction sets presented in this study demonstrate how the logic server can be configured to respond to collected data with location-contingent or time-contingent prompts.”  In addition, we have provided a further description of the methodological improvements made by the HEALTH Platform as compared to previous tools:  “The HEALTH Platform improves upon other GEMA tools by providing a flexible system architecture that supports a wide range of data collection methods, the means to vary prompts using complex location and/or time-based criteria, and the capability to adjust system instruction sets at an individual participant level or in response to participant behaviour. The logic server can use complex geometries as part of the geofencing evaluation process in the system instruction sets, as compared to more traditional proximity or buffer-based approaches to the triggering of location-contingent prompts in other GEMA studies. In addition, the logic server can relate individual geographic features and their attributes to specific prompts or recorded data, instead of relying on post-data collection analyses, substantially increasing the nuance of logic applied in the system instruction sets, and spatiotemporal accuracy of collected data. In short, the HEALTH Platform is a transformative improvement for the conduct of GEMA studies.” |
| R1.4 | Distinguish conclusion, discussion and future work section The current ending discussion sections include some discussion around best practice of GEMA studies, various existing challenges and how the HEALTH platform could potentially contribute to addressing those challenges, and general considerable advantages of HEALTH platform. But everything is blended. A more clear structure with the key findings of conducting ParkSurvey with the HEALTH platform, the current contribution how HEALTH platform is improving the GEMA studies, and areas for future improvement of HEALTH platform would significantly improve readability and uptake by readers. | We have reorganized the Discussion section (4) to separate out methodological findings directly related to the ParkSeek study, and findings related to the HEALTH Platform in general. |
| R1.5 | Some other minor comments:  a. What kind of Privacy and Security standard the platform is sticked to? HIPAA/GDPR? | We have provided additional description at approximately Line 296 of how the HEALTH Platform addresses common privacy and security standards:    “The HEALTH Platform’s architecture and participant data controls conform to the privacy and security standards found in major national legal frameworks. These include the United States of America’s *Health Insurance Portability and Accountability Act* Privacy Rule and Security Rule, the European Union’s *General Data Protection Regulation* seven protection and accountability principles, and Canada’s *Freedom of Information and Protection of Privacy Act* (Ontario) Collection Limitation principle and Data Security standards. The HEALTH Platform implements these frameworks by only collecting and storing data that is absolutely necessary for the operation of the platform, centralizing the platform to use only on-premises infrastructure exclusively controlled by the researchers, seeking informed and ongoing consent from all participants through the use of the invite codes, and providing participants with detailed controls over the data collection mechanisms and the ability to withdraw their data from the platform.” |
| R1.6 | b. According to the paper, “Any functionality available within Esri’s ArcGIS Enterprise, software ecosystem can be configured within the HEALTH Platform’s monitor server.” What kind of configuration would it need, coding/pseudo-coding? Any limit of relying on Esri’s ArcGIS Enterprise? | As noted in our reply to R1.2, the software ecosystem is a low-code environment. The use of Esri’s ArcGIS Enterprise and the Expo Application Service has no unique limitations as compared to other commercial software, with Esri’s software being freely available to most academic researchers, while substantially reducing the administrative, financial, and technical overhead associated with traditional bespoke software development. |
| R2.1 | Review for The Healthy Environments and Active Living for Translational Health (HEALTH) Platform: A smartphone-based platform for geographic ecological momentary assessment research | We thank the reviewer for their feedback and detailed copy-editing of the manuscript. We have provided detailed responses to their feedback below. |
| R2.2 | Title: These are my concerns:  - Try to capitalize the first letter of each word of the title to make it coherent.  - the word “platform” is used twice and should be revised to avoid repetition.  - It combines many academic keywords (e.g., ecological momentary assessment, geographic, translational health), which may reduce clarity and immediate comprehension.  - Shorten the title to focus on the scope and impact.  - Examples: The HEALTH Platform: A Smartphone-Based Tool for Geographic Ecological Momentary Assessment or The HEALTH Platform for Translational Health: Smartphone-Based Geographic Ecological Momentary Assessment | We have reduced the repetition of “platform” in the title. We think the current title capitalizing the elements of the HEALTH Platform functions well, and the current words are an appropriate description for scientific publication. |
| R2.3 | The abstract, these are my comments:  - The abstract contains long sentences and overuses technical phrases, making it harder for a general audience to absorb.  - Results from the illustrative study are vague; no quantitative findings or sample characteristics are provided.  - The statement “improvements upon existing GEMA platforms” is too general—what specific enhancements were made (e.g., geofencing precision, data flexibility)? | We have made edits to the abstract based on the reviewer’s direct suggestions, as well as our own improvements to provide a clearer summary of the study. The purpose of the manuscript is to illustrate the HEALTH Platform’s capabilities, not provide empirical findings of the demonstrative study of park exposure and health, so inclusion of results in the abstract is not appropriate. |
| R2.4 | Line 20–21: “Smartphones are an increasingly popular delivery mechanism...” Suggest: “Smartphones have become a widely used tool for delivering digital health interventions and conducting observational research.” | Suggested edit made in manuscript. |
| R2.5 | Line 22–23: “which can be extended by incorporating the collection of participant location data...” Suggest: “which can be enhanced by collecting participant location data using built-in smartphone technologies.” | Suggested edit made in manuscript. |
| R2.6 | Line 24: “customizable, geographically appropriate software” Suggest: “customizable software capable of supporting geographically explicit research.” | Suggested edit made in manuscript. |
| R2.7 | Line 29–30: “without the need for time-consuming updates...” Suggest: “without requiring time-consuming updates to participants’ devices.” | Suggested edit made in manuscript. |
| R2.8 | Line 31–32: “This study demonstrates the HEALTH Platform’s improvements upon existing GEMA software platforms.” → Suggest: “This study illustrates how the HEALTH Platform improves upon existing GEMA software by offering greater customization and real-time flexibility.” | Suggested edit made in manuscript. |
| R2.9 | Line 34–35: “Overall, we find that the HEALTH Platform is a flexible mobile phone application...” Suggest: “Overall, the HEALTH Platform offers a flexible solution for implementing GEMA in digital health research.” | Suggested edit made in manuscript. |
| R2.10 | The introduction:  • The section is quite long and could benefit from clearer sectioning or condensation of overlapping ideas (e.g., multiple discussions of smartphone EMA vs pre-digital EMA). | We have added additional headings to better organize this section of the manuscript. We have not condensed the information as we think it is important and necessary background to provide the justification for the development of the HEALTH Platform. |
| R2.11 | The research objective (lines 161–164) comes very late. It would be more effective to briefly state the study’s purpose at the beginning and then expand upon it later. | We think the study’s purpose is appropriately stated in the manuscript at the end of the introduction, logically leading from the background provided about the topic. |
| R2.12 | Some claims (e.g., on technical limitations of GEMA platforms) heavily rely on the same few sources ([24], [25], [40]), which weakens the perception of broad evidence. | We think these references show broad evidence as they are the two most recent systematic reviews of GEMA studies, with each summarizing tens of studies that have used this method. |
| R2.13 | While limitations of existing GEMA platforms are described, the authors could be more explicit about why addressing these limitations matters for scientific or population health outcomes. | We have emphasized the contributions of GEMA to improving population health at approximately Line 152: “In summary, these improvements from GEMA deliver more reliable and valid measures of health behaviours and outcomes, resulting in more precise interventions for population health.” |
| R2.14 | Line 60: “has become almost universally accessible to the general population in developed nations.” Suggest: “is now widely accessible in most developed countries.” | Suggested edit made in manuscript. |
| R2.15 | Line 65: “observe communicable disease patterns [13,14, and promote sustained change…” Correction: Missing closing bracket: [13,14] | Suggested edit made in manuscript. |
| R2.16 | Line 84–85: “addressed many of these concerns about recall and desirability bias…” Suggest: “helped mitigate concerns related to recall and desirability bias…” | Suggested edit made in manuscript. |
| R2.17 | Line 87–88: “based on temporal context, that is participants respond…” Suggest: “based on temporal context—that is, participants respond…” | We have made an alternative edit to the manuscript to improve the clarity of these sentences. |
| R2.18 | Line 110–111: “theoretically informed by the social-ecological model of health, where…” Suggest: “… model of health, in which individual behaviors…” | Suggested edit made in manuscript. |
| R2.19 | Line 142–161 (Motivation paragraph): Overly dense. This could be split into two paragraphs: one detailing the problems with existing GEMA tools and a second outlining the authors’ solution and objectives. | We agree and have split this section into two paragraphs. |
| R2.20 | Line 157: “…while also addressing the current technical limitations found in the digital health research area.” Suggest: “…while addressing key technical limitations currently faced in digital health research.” | Suggested edit made in manuscript. |
| R2.21 | Materials and Methods:  Some sections (especially on architecture and software libraries) may overwhelm readers without computer science backgrounds. Consider moving these to an appendix or supplementary material. | As a methodological publication, the description of the software and system architecture is an important inclusion in the main text. |
| R2.22 | The ParkSeek study lacks information on sample size, demographics, adherence rates, and outcomes, which are crucial for assessing feasibility and generalizability. • The authors should acknowledge technical or practical limitations (e.g., battery drain, GPS inaccuracies, reliance on Esri licenses). | We have clarified the study’s purpose as purely methodological at approximately Line 347:  “The purpose of the following case study is to illustrate the potential of the HEALTH Platform for GEMA research. This example is not intended to provide any actionable and meaningful findings relevant to the hypothesized relationship between park exposure and health. Instead, the following sections provide methodological findings that exemplify the HEALTH Platform’s improvements upon past (G)EMA methods. Moreover, the system instruction sets presented in this study demonstrate how the logic server can be configured to respond to collected data with location-contingent or time-contingent prompts.”  We report on these technical and practical limitations in multiple sections of the manuscript. |
| R2.23 | No benchmarks or error rates are reported for prompt delivery success, location accuracy, or video data quality. | No issues were encountered with the delivery success of the prompts. Location accuracy is automatically handled by the platform. Videos were recorded in the same format to avoid data quality issues. |
| R2.24 | Only one pilot study is provided, with no comparison to other EMA/GEMA tools or platforms. | As stated previously, that is the intended purpose of this manuscript. Later studies may provide more direct comparisons to other platforms in controlled data collection environments. |
| R2.25 | Repetitive phrasing: “The HEALTH Platform app…” and “logic server…” appear excessively. Use pronouns or merge sentences for variety. | We have reviewed uses of these phrases, substituting pronouns where appropriate. |
| R2.26 | Redundant language: “A flexible and secure software system…” and later “flexible unified software platform…” try to revise for variation and conciseness. | We have replaced this redundant language for clarity. |
| R2.27 | Improper punctuation and spacing: Line 65: “promote sustained change in health behaviours [3,15,16].” Ensure proper spacing and citation bracket closure (which was missing earlier). | Suggested edit made in manuscript. |
| R2.28 | Overuse of passive voice: E.g., “data is transmitted,” “survey is prompted,” “responses are recorded.” Consider active voice where clarity permits | We have reviewed passive and active voice use to improve clarity in the manuscript. |
| R2.29 | Lines 243–249 and 295–307 could be broken into shorter sentences to improve readability. | We have made changes where appropriate to these sentences |
| R2.30 | Ensure consistent use of past tense when describing completed studies (e.g., ParkSeek), and present tense when referring to platform functionality. | We have reviewed the tenses used throughout the manuscript, making corrections where needed. |
| R2.31 | Results:  Only 38 participants met the inclusion threshold out of 1,379 contacted. This significantly limits the generalizability and statistical power of the results. | Not the purpose of this manuscript. Future studies will require a larger sample size to be generalizable. |
| R2.32 | The section does not report findings on how park exposure influenced well-being or sleep scores, even descriptively. | Not the purpose of this manuscript, as stated earlier. |
| R2.33 | Race, socioeconomic status, education level, or geographic region (urban/rural) are underreported despite being highly relevant. | Not the purpose of this manuscript, as stated earlier. |
| R2.34 | No t-tests, regression models, or other statistical analyses were presented to assess adherence differences or behavioral outcomes. | Not the purpose of this manuscript, as stated earlier. |
| R2.35 | While video submission rates are mentioned, no content analysis, coding, or qualitative findings are discussed. | Not the purpose of this manuscript, as stated earlier. |
| R2.36 | Line 311:“whereby the outcome measure for participants was measured outside…” Suggest: “in which the outcome measure was collected outside…” | Suggested edit made in manuscript. |
| R2.37 | Line 314: “therefore being affected…” Suggest: “and were therefore affected…” | Suggested edit made in manuscript. |
| R2.38 | Lines 324–326: Redundant: “Consent was obtained in two stages... participants indicated their consent...” Suggest: Combine into one sentence for flow: “Consent was obtained in two stages, including explicit agreement to the location logging function.” | We have made an alternative edit to the manuscript to improve the clarity of these lines. |
| R2.39 | Lines 329–344: This paragraph could be split into two: one for the onboarding/survey process and one for post-study assessment. | Suggested edit made in the manuscript. |
| R2.40 | Lines 380–392: The phrase “participants were also prompted to…” is repeated. Suggest: Vary the structure for better readability. | We have made an alternative edit to the manuscript to improve readability. |
| R2.41 | Table 1 title: Title should specify that the table includes both adherence and latency statistics, e.g., “Participant Adherence and Response Latency by Prompt Type in the ParkSeek Study” | Suggested edit made in manuscript. |
| R2.42 | Line 417: “...a video was submitted 23% of the time” Suggest: “Videos were submitted in 23% of engagement-type prompt responses. | We have made an alternative edit to the manuscript to improve the clarity of these lines. |
| R2.43 | Discussion:  The authors could expand on strategies for improving participant engagement, especially in low adherence environments (e.g., parks). Try to include more concrete future recommendations (e.g., gamification, tailored incentives). | We have improved the discussion to engage with the detailed points of revision raised below. |
| R2.44 | Low adherence to video responses undermines this proposed advantage. Suggestion: Propose detailed implementation of “photovoice” training or alternative non-intrusive context capture methods. | This is already stated in the manuscript as a potential solution to the low adherence issue with video-based prompts. |
| R2.45 | Add a sentence indicating what features are already operational and which are prospective. | We have clarified the features that are hypothetical versus already implemented in the platform. |
| R2.46 | Consider including a brief ethical discussion on audiovisual data usage, especially in public settings. - Line 434: “only 40% achieved an acceptable level of adherence to the study protocol.” Consider: “Only 40% of participants adhered sufficiently to the study protocol.” | Suggested edit made in manuscript. |
| R2.47 | Line 437: “adherence with responding to prompted assessments” Should be: “adherence to prompted assessments” | Suggested edit made in manuscript. |
| R2.48 | Line 445: “most complex and/or longest questionnaires” Prefer: “most complex or longest questionnaires” | Suggested edit made in manuscript. |
| R2.49 | Line 454: “there is considerably low uptake” Correct to: “there is considerably low uptake” → “uptake is considerably low” | Suggested edit made in manuscript. |
| R2.50 | Line 463: “models of behaviour change and personalized interventions.” Parallel structure might benefit from: “behavior change models and personalized interventions” | Suggested edit made in manuscript. |
| R2.51 | Line 474: “may not be an accurate representation of their normal behaviours” suggests: “may not accurately represent their typical behavior” | Suggested edit made in manuscript. |
| R2.52 | Line 492: “participants were not given any guidance on how to record videos” suggests: “participants received no guidance on recording videos” | Suggested edit made in manuscript. |
| R2.53 | Line 517: “that needs to include” Improve to: “that require inclusion of” | Suggested edit made in manuscript. |
| R2.54 | Line 529: “ensures flexibility to accommodate all potential research designs” More precise, “provides flexibility to support a wide range of research designs” | Suggested edit made in manuscript. |
| R2.55 | Line 551: “highly accessible and feasible for use among a range of populations.” Rephrasing suggest: “feasible and accessible across diverse populations.” | We have made an alternative edit to the manuscript to improve the clarity of these lines. |
| R2.56 | References:  Reference numbers [e.g., 12, 61] include the journal name but not consistently in italics. Standard referencing style (e.g., AMA, APA, Vancouver) should be applied uniformly. | Our understanding of the journal’s referencing guidelines is that journal titles are not italicized. We have followed the journal’s standard citation format, which appears to follow a hybrid of AMA and Vancouver. |
| R2.57 | There are inconsistencies in journal abbreviations (e.g., JMIR mHealth uHealth vs. J Med Internet Res vs. PLOS Digit Health). Consider standardizing based on the target journal's guidelines. | We have used the NLM’s journal abbreviations. Any perceived inconsistencies are based on any inconsistencies that may be found in the NLM database. |
|  | Ref. 61 (O’Bright K) is a valuable addition but should be clearly marked as a thesis and cross-checked for DOI or permanent repository link format. | The citation is clearly marked as “MES Thesis” and uses the University’s recommended linking format for theses. |
| R2.58 | References 2, 5, 6, and 15 are all meta-reviews on mobile interventions—consider streamlining to reduce overlap unless each is cited for a distinct contribution. • References such as [17], [20], [21] (Stone, Mehl, Silvia) are essential and well-cited; however, ensure that the main text leverages their methodological frameworks explicitly. | These meta-reviews are cited to provide readers direction to sources of a wide-range of EMA and mobile health studies. In addition, we have deployed other references in an appropriate manner that matches their specific contribution to our provision of supporting information throughout the manuscript. |
| R2.59 | Ref. 4: The citation of the Ericsson Mobility Report includes a URL that may need formatting as per the journal style (e.g., accessed date placement). | As per the citation standards, the accessed date is the “Cited [date]” for this reference. |
| R2.60 | Ref. 29: Appears to list “Pred A” twice; double-check for typographical duplication in author names. | There is only one Pred A. (1977) citation in the reference list. |
| R2.61 | Ref. 56 and 57: The authors “Astell-Burt et al.” and “Barber et al.” appear in the same paragraph in the discussion—ensure the narrative distinguishes their contributions in-text. | References have been split in this sentence to clarify their contributions in the text. |
| R3.1 | This manuscript introduces the HEALTH Platform, a customizable smartphone-based system for implementing geographically explicit ecological momentary assessment (GEMA) in digital health research. The authors present the platform's design, architecture, and capabilities, and illustrate its functionality through the ParkSeek study, which investigates associations between park exposure and well-being. The study is timely and addresses a clear gap in the digital health landscape, particularly in the availability of flexible, privacy-preserving, and scalable tools for GEMA research. The manuscript is well-structured and generally clear, although there are areas that would benefit from further clarification and discussion. | We thank the reviewer for their positive review of our manuscript. |
| R3.2 | Pros:  - Platform fills a critical void in GEMA tools, offering a flexible, secure, and cross-platform solution that supports audiovisual data, dynamic geofencing, and real-time logic control.  - The authors present a unique contribution that goes beyond traditional EMA methods. | We agree with the reviewer that these are strengths of our manuscript. |
| R3.3 | Cons:  - While the manuscript notes adherence challenges, the authors can discuss usability issues, including qualitative feedback from participants or a user study. | We thank the reviewer for providing suggestions to improve our manuscript. We provide detailed responses to their feedback below. |
| R3.4 | More insight into why participants avoided video recordings and how this might be addressed (e.g., training, incentives, privacy messaging) would strengthen the discussion. | Further commentary on the video recordings is beyond the remit of this manuscript, as a purely methodological study. Future studies with a larger sample size and directly related protocol to evaluating the video-based prompts is an ideal next step for the platform’s development and evaluation. |
| R3.5 | How can the platform made more scalable? The authors can also discuss the potential barriers to adoption of this system in public health practice or clinical trials. | We think the HEALTH Platform is already highly scalable as compared to other GEMA software tools. We have commented on the need to evaluate the HEALTH Platform in clinical trial and public health practice settings at approximately Line 670:  “Future studies may wish to more comprehensively evaluate the performance of the HEALTH Platform as compared to other software platforms when used by the same group of participants. Implementation of the HEALTH Platform in a clinical trial or public health practice setting are also desirable areas for future studies. These studies could be used to identify and address barriers to implementation such as technological adoption, patient and provider data privacy concerns, and the scalability of the system to multiple treatment conditions.” |
| R3.6 | Maybe elaborate on built-in data analysis pipelines or tools available to users through the monitor server. | We have clarified that the monitor server is practically limitless in its capabilities at approximately Line 198. |
| R3.7 | Comparison with Existing Tools: A concise table or section comparing the HEALTH Platform with other GEMA-capable apps (e.g., Itinerum, Beiwe, or bespoke REDCap-GIS integrations) would contextualize its novelty more clearly. | We have made an addition at approximately Line 163 to address this request. |
| R3.8 | The description of prompt delivery logic (especially around “park engagement follow-up”) can be overly technical in places. Consider simplifying or visualizing the logic to improve reader comprehension. This might increase the reproducibility of the model. | We have clarified the description of the prompts throughout the manuscript. |
| R3.9 | Minor comments:  Check that the formatting of figures and citations are consistent. An external check is needed. | We have reviewed all figures and citations to ensure consistent formatting. |
| R3.10 | Consider including example visualizations from the dashboard interface. | Figure 3 already provides an example visual of the dashboard interface. |
| R3.11 | Is the app is open-source or available to other researchers and under what licensing terms. | The app and platform are not available open-source at this time, as it makes use of commercially licensed software components and is still in continual development. Interested researchers may contact the research team for further information about making use of the HEALTH Platform. |
